# Rise of Regional Anesthesia in Emergency Medicine: A Bibliometric Analysis of Global Research Trends and Collaborative Networks

**DOI:** 10.7759/cureus.113510

**Published:** 2026-07-28

**Authors:** Abhijit Nair, Badar Al Hamrashdi, Tuhin Mistry, Ahmed Al-Jabri

**Affiliations:** 1 Anesthesiology, Ibra Hospital, Ibra, OMN; 2 Emergency Medicine, Rustaq Hospital, Rustaq, OMN; 3 Anesthesiology, Ganga Medical Centre and Hospitals, Pvt. Ltd, Coimbatore, IND; 4 Emergency Medicine, Ibra Hospital, Ibra, OMN

**Keywords:** bibliometric analyis, emergency medical service, emergency medicine and trauma, nerve block, regional anesthesia

## Abstract

Regional anesthesia (RA) is increasingly utilized in emergency departments (EDs) as an opioid-sparing strategy for acute pain management. However, the global research landscape, collaboration patterns, and thematic evolution of RA in emergency medicine (EM) remain poorly characterized. This study aimed to map the development of this literature and identify research priorities relevant to anesthesiologists involved in acute pain, trauma care, education, and multidisciplinary emergency services. This bibliometric analysis was conducted using the Scopus records from the database's inception to January 2026. Scopus was selected because it provides broad multidisciplinary journal coverage together with structured citation, affiliation, and author metadata required for bibliometric mapping. A search query focused on "emergency medicine" and "regional anesthesia" was applied. Bibliometric indicators, including annual scientific production, citation patterns, country contributions, journal productivity, co-authorship, co-citation, and keyword co-occurrence, were analyzed using the bibliometrix package version 5.2.1 (Massimo Aria, Federico II University, Naples, Italy) in RStudio version 2024.12.1 (Posit Software, PBC, Boston, MA, USA) through Biblioshiny (the web interface of bibliometrix), and VOSviewer version 1.6.20 (Centre for Science and Technology Studies, Leiden University, Netherlands). A total of 232 documents published between 1985 and 2026 across 79 sources were included. The annual growth rate was 4.47%, with a mean citation count of 12.2 per document. Research output increased significantly after 2015. The United States was the leading contributor in both publication volume and total citations (n=1297). The American Journal of Emergency Medicine (AJEM) and the Journal of Emergency Medicine (JEM) were the most productive sources. Brachial plexus block, erector spinae plane block, and fascia iliaca block were the most frequently studied interventions. International co-authorship was relatively low at 9.9%. Research on RA in EM has grown steadily, particularly during the past decade, but remains geographically concentrated and characterized by limited international collaboration. The prominence of ultrasound-guided peripheral nerve and fascial plane blocks reflects sustained scientific attention rather than proof of clinical superiority. For anesthesiology, these trends highlight opportunities to strengthen multidisciplinary training, standardize outcome reporting, and develop multicenter studies that evaluate safety, implementation, and patient-centered outcomes in emergency care.

## Introduction and background

Regional anesthesia (RA) has emerged as an important component of acute pain management in the emergency department (ED), providing site-specific analgesia while reducing reliance on systemic opioids [1]. The growing emphasis on opioid-sparing strategies in emergency medicine (EM) has intensified interest in ultrasound-guided RA. These techniques are used for painful conditions such as fractures, dislocations, and acute soft-tissue injuries [2]. Advances in point-of-care ultrasound (POCUS) and its integration into EM training have also facilitated the performance of RA by emergency physicians. This development is directly relevant to anesthesiologists because it expands RA techniques beyond the operating theatre and creates a need for shared standards in training, governance, safety, and complication management [3,4]. 

Despite these developments, the utilization of RA in emergency settings remains variable. This inconsistency is often influenced by factors such as specialized training, resource availability, and varying institutional protocols. While clinical studies have demonstrated that nerve blocks significantly improve analgesia and reduce opioid-related side effects, the global research activity and scientific evolution of this field have not yet been systematically evaluated.

Bibliometric analysis is a quantitative method used to evaluate scientific output, research trends, and collaboration networks within a defined field [5]. It identifies influential publications, major contributors, and emerging themes, thereby helping to reveal gaps in research organization and knowledge production. Although bibliometric approaches have been applied to several areas of anesthesia and EM, a focused analysis of RA in the ED is lacking. Therefore, this study aimed to analyze publication trends, authorship patterns, collaborative networks, citation structure, and thematic evolution in this field. In addition to describing the literature, we sought to determine how these patterns may inform research priorities, multidisciplinary training, and the future contribution of anesthesiology to emergency pain care.

## Review

Methods

This study was conducted as a bibliometric analysis, an observational research method used to evaluate scientific output, publication trends, and collaboration patterns within a specific research field.

Data Source and Search Strategy

Bibliometric data were retrieved from Scopus. Scopus was selected as the sole source because it provides broad multidisciplinary coverage of peer-reviewed journals and structured metadata for citations, authors, affiliations, institutions, and countries, which are required for reproducible science mapping. Use of a single database also avoids duplicate records and inconsistencies that can arise when metadata from multiple databases are merged. The search was conducted from database inception to January 2026 by applying the following query to titles, abstracts, and keywords: TITLE-ABS-KEY (("emergency department" OR "emergency medicine" OR "emergency ward")) AND TITLE-ABS-KEY (("regional anesthesia" OR "regional anaesthesia" OR "peripheral nerve block" OR "fascial plane block")). No restrictions were placed on language or study design. All retrieved records were manually screened, and records not related to RA in emergency settings were excluded. A total of 232 documents were included in the final analysis. Although Scopus was methodologically suitable for the planned analyses, publications indexed exclusively in PubMed/Medical Literature Analysis and Retrieval System Online (MEDLINE), Web of Science, or regional databases were not captured.

Study Protocol and Visualization

The retrieved records were exported in a structured format and analysed using the bibliometrix package version 5.2.1 (Massimo Aria, Federico II University, Naples, Italy) in RStudio version 2024.12.1 (Posit Software, PBC, Boston, MA, USA) through Biblioshiny (the web interface of bibliometrix), and VOSviewer version 1.6.20 (Centre for Science and Technology Studies, Leiden University, Netherlands) [6,7]. Descriptive indicators included annual scientific production, relevant sources, authorship patterns, citation impact, and country contributions. Co-authorship, co-citation, and keyword co-occurrence analyses were used to examine collaboration structures and thematic relationships. Thresholds were applied to network maps to improve readability and interpretability.

Outcome Measures

The primary outcomes were annual publication trends, citation patterns, productive authors and journals, and country-level scientific output. Secondary outcomes were collaboration networks, co-citation relationships, and keyword-frequency distributions. These outcomes were interpreted as indicators of research activity and thematic attention; they were not used to infer the clinical efficacy or superiority of any regional anesthetic technique.

Statistical Analysis

Data analysis used descriptive bibliometric indicators. The bibliometrix package was used to calculate annual growth rate, mean citations per document, authorship indicators, and collaboration indices. VOSviewer was used for network visualization with association-strength normalization. Before thematic analysis, spelling variants and synonymous terms were standardized, including "regional anesthesia/regional anaesthesia" and related ultrasound terminology. Generic indexing terms were excluded from thematic co-occurrence interpretation. For transparency, the raw rank-frequency dataset used for Zipf analysis retained all indexed terms, including "human"; such generic terms were not interpreted as clinical research themes. Keyword rank-frequency data were plotted on logarithmic axes to assess conformity with Zipf's law [8]. The plot was generated in R using the ggplot2 package (developed by Hadley Wickham) and the ggrepel package (developed by Kamil Slowikowski).

Ethical Approval and Consent

This bibliometric analysis was based exclusively on published bibliographic records retrieved from Scopus and did not involve patients, identifiable human data, medical records, biological samples, or any clinical intervention. Hence, formal ethics approval and patient consent were not applicable.

Results

Publication Trends and Data Characteristics

The initial search identified 533 records. After deduplication and manual screening for relevance, 232 documents published between 1985 and 2026 were included. These documents spanned 79 sources and cited 1,629 references. The annual publication growth rate was 4.47%, the mean citation count was 12.2 per document, and the mean document age was 7.49 years. The dataset contained 1,033 authors, with a mean of 9.01 co-authors per document. International co-authorship occurred in 9.9% of documents. A summary of dataset characteristics and document types is provided in Table 1.

**Table 1:** Summary of the analysis

Description	Results
MAIN INFORMATION ABOUT DATA	
Timespan, years	1985-2026
Sources (journals, books, etc), n	79
Documents, n	232
Annual growth rate, %	4.47
Document average age, years	7.49
Average citations per document	12.2
References, n	1629
DOCUMENT CONTENTS	
Keywords plus (ID), n	2060
Author's keywords (DE), n	396
AUTHORS	
Authors, n	1033
Authors of single-authored documents, n	0
AUTHORS COLLABORATION	
Single-authored documents, n	0
Co-Authors per documents	9.01
International co-authorships, %	9.914
DOCUMENT TYPES	
Articles, n	167
Conference papers, n	2
Editorials, n	7
Letters, n	16
Notes, n	2
Reviews, n	37
Short surveys, n	1

**Figure 1:**
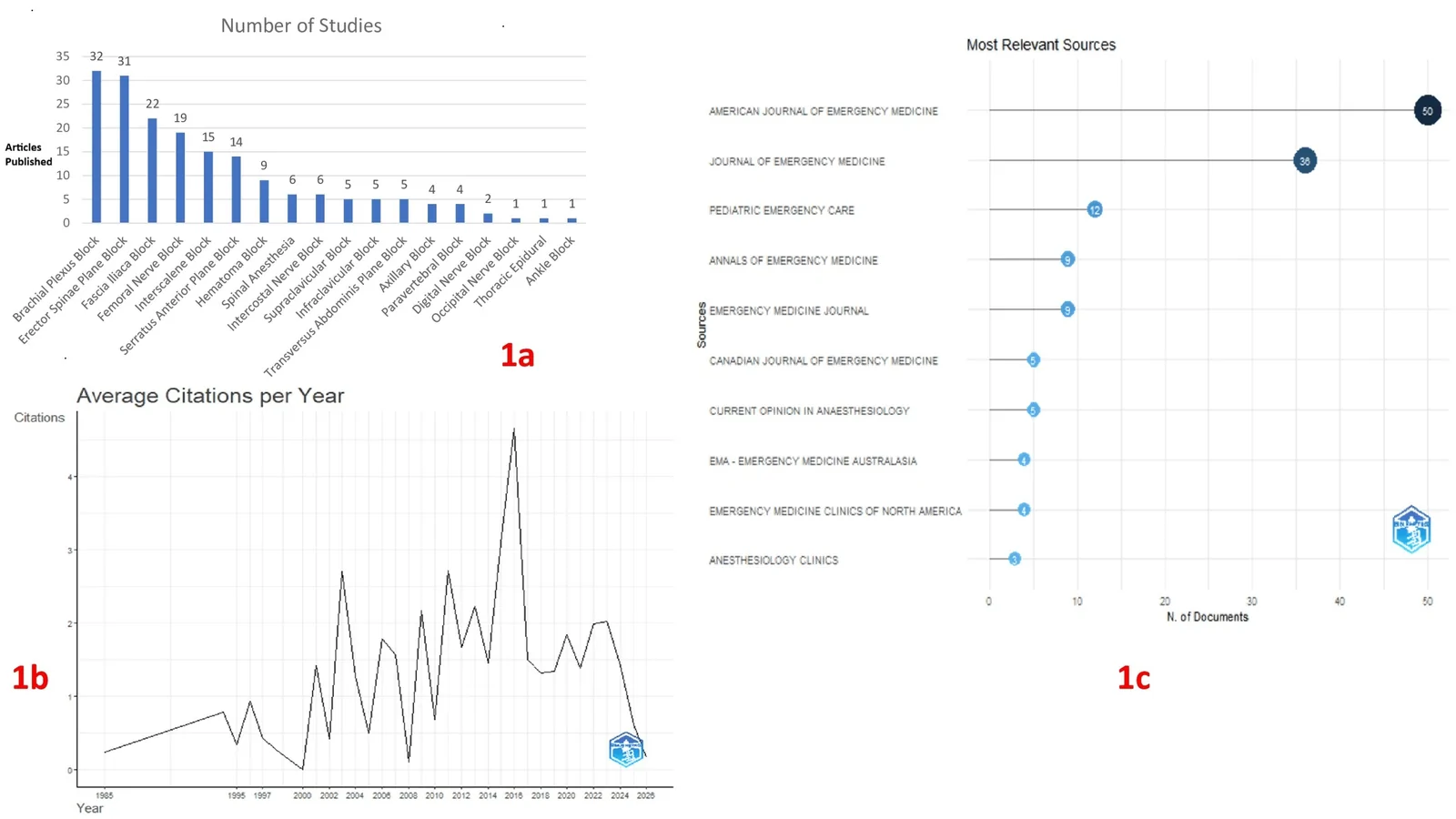
Publication trends, data characteristics, journal productivity, and impact a. Bar diagram showing the distribution of studies based on the various types of regional anesthesia blocks; b. Line graph depicting average citations per year since inception; c. Lollipop chart showing the most relevant journals based on the number of articles published

Regarding specific interventions, the brachial plexus block was the most frequently studied (n=32), followed by the erector spinae plane block (n=31) and fascia iliaca block (n=22) (Figure 1a). Scientific production remained modest until 2015, after which a marked increase was observed, peaking in 2024-2025 (Figure 1b).

Journal Productivity and Impact

**Figure 2:**
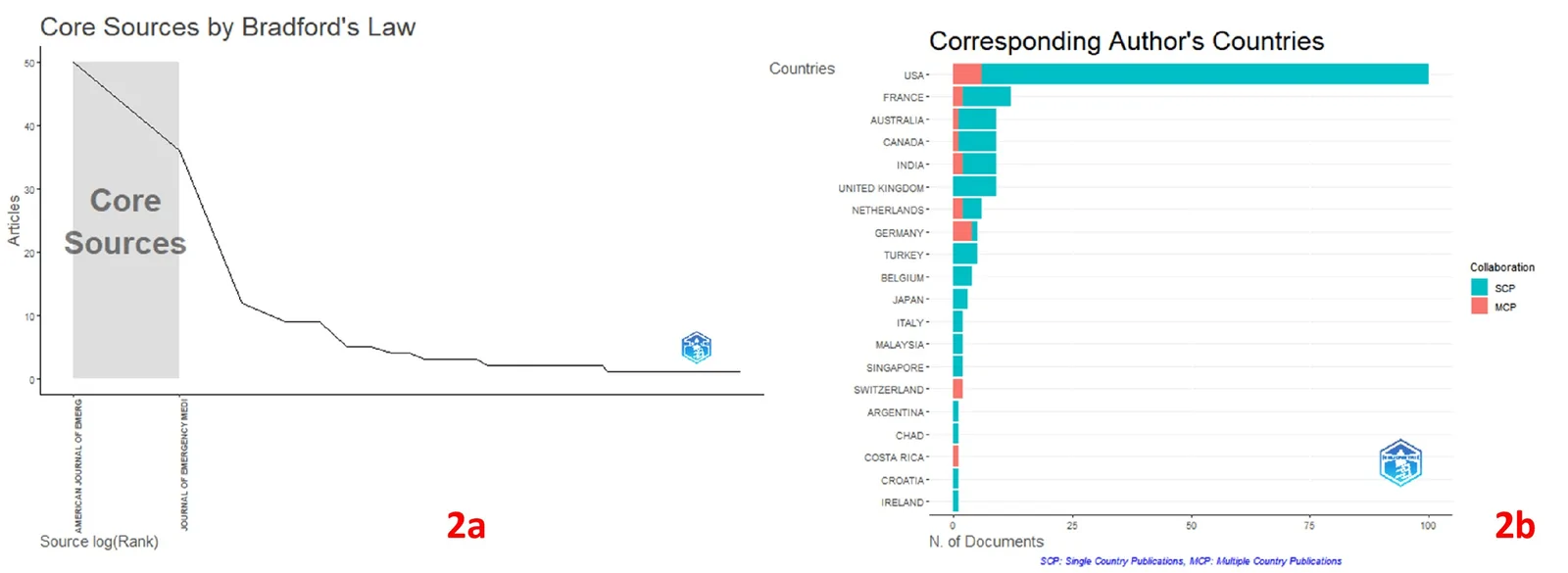
Figures showing the core sources according to Bradford's Law and the corresponding authors' countries. a. The figure illustrates the distribution of various journals and identifies the core source based on Bradford's Law. The x-axis shows source rank on a logarithmic scale, and the y-axis shows the number of articles. b. Geographical distribution of research production and research collaboration. SCP: single-country publications, MCP: multiple-country publications

The American Journal of Emergency Medicine (AJEM) was the most productive venue with 50 documents, followed by the Journal of Emergency Medicine (JEM) with 36 (Figure 1c). Bradford’s Law analysis identified a small core nucleus of journals driving the field, with AJEM and JEM serving as the primary sources (Figure 2a) [9].

Author and Publication Impact

**Figure 3:**
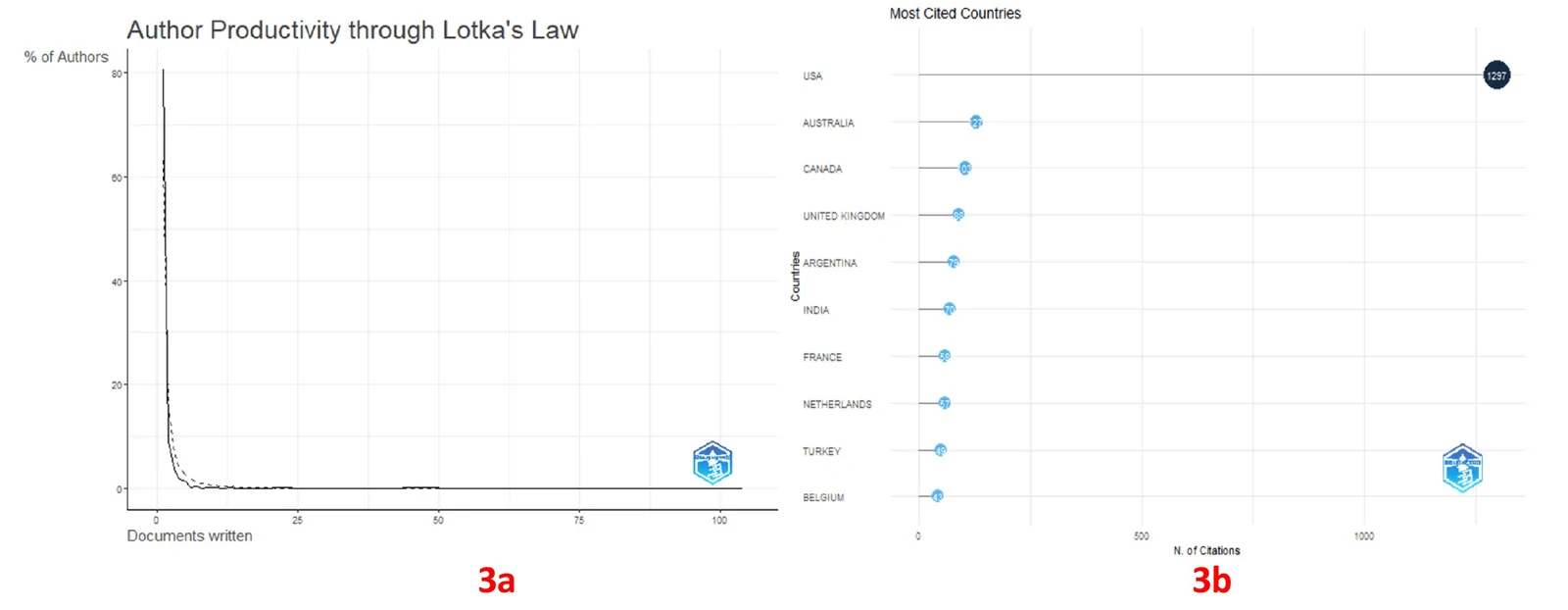
Author's productivity and countries with the highest impact a. Chart visualizing author productivity through Lotka's Law. The x-axis shows the number of documents published per author, and the y-axis shows the percentage of authors. b. Lollipop chart showing countries with the highest impact (Y-axis) based on the total number of citations (X-axis)

A total of 1,033 authors contributed to the literature. The most cited publication was the work by Krauss et al. (2016) in The Lancet (181 citations) [10], followed by Cao et al. (2015) [11], and Beaudoin et al. (2013) [12]. Table 2 provides a comprehensive summary of the top ten authors, the journals, and the number of citations received [10-19]. Analysis of corresponding authors revealed that most research is driven by single-country publications (SCP), with the United States and Canada exhibiting the highest absolute numbers of multiple-country publications (MCP) (Figure 2b). Lotka’s Law analysis indicated that a small subset of prolific authors accounts for a disproportionate amount of the total output (Figure 3a) [20]. In the co-authorship network, Michael Shalaby was the most prolific author (15 documents), while Arun D. Nagdev had the highest total citation count (n=176) (Table 3). The Department of Emergency Medicine at Rush University Medical Center was the most cited organization (Table 4).

**Table 2:** Summary of the top 10 authors with the journal's name and the number of citations received JEM: Journal of Emergency Medicine, AJEM: American Journal of Emergency Medicine

Author/year	Journal	Number of citations
Krauss et al., 2016 [10]	Lancet	181
Cao et al., 2015 [11]	JEM	129
Beaudoin et al., 2013 [12]	Annals of Emergency Medicine	127
Godoy Monzon et al., 2007 [13]	JEM	90
Haines et al., 2012 [14]	JEM	85
Todd KH, 2017 [15]	Pain Therapy	79
Migita et al., 2006 [16]	The Archives of Pediatrics and Adolescent Medicine	67
Yen et al., 2003 [17]	Annals of Emergency Medicine	65
O'Connor et al., 2011 [18]	Annals of Emergency Medicine	64
Long et al., 2022 [19]	AJEM	60

**Table 3:** Details of co-authorship and countries generated using the VOSviewer

Co-authorship and authors		
Author	Documents	Citations
Richard J. Gawel	9	29
Andrew J. Goldsmith	9	69
Michael A. Gottlieb	7	83
Andrew A. Herring	7	125
Jeffrey A. Kramer	5	12
Daniel Mantuani	5	45
Chitta Ranjan Mohanty	5	66
Arun D. Nagdev	14	176
Rakesh Vadakkethil Radhakrishnan	5	66
Michael Shalaby	15	64
Robert T. Stenberg	5	13

**Table 4:** Details of co-authorship and organizations generated using the VOSviewer

Co-authorship and organizations		
Organization	Documents	Citations
College of Nursing, All India Institute of Medical Sciences, Bhubaneswar, India	5	66
Department of Emergency Medicine, Harvard Medical School, Boston, United States	5	25
Department of Emergency Medicine, Highland General Hospital, Oakland, United States	11	94
Department of Emergency Medicine, Hospital of the University of Pennsylvania, Philadelphia, United States	8	24
Department of Emergency Medicine, Mount Sinai Medical Center, Miami Beach, United States	6	37
Department of Emergency Medicine, Rush University Medical Center, Chicago, United States	7	83
Department of Trauma and Emergency, All India Institute of Medical Sciences, Bhubaneswar, India	5	66
University of California, San Francisco (UCSF), School of Medicine, San Francisco, United States	5	60

Geographical Distribution and Collaboration

**Figure 4:**
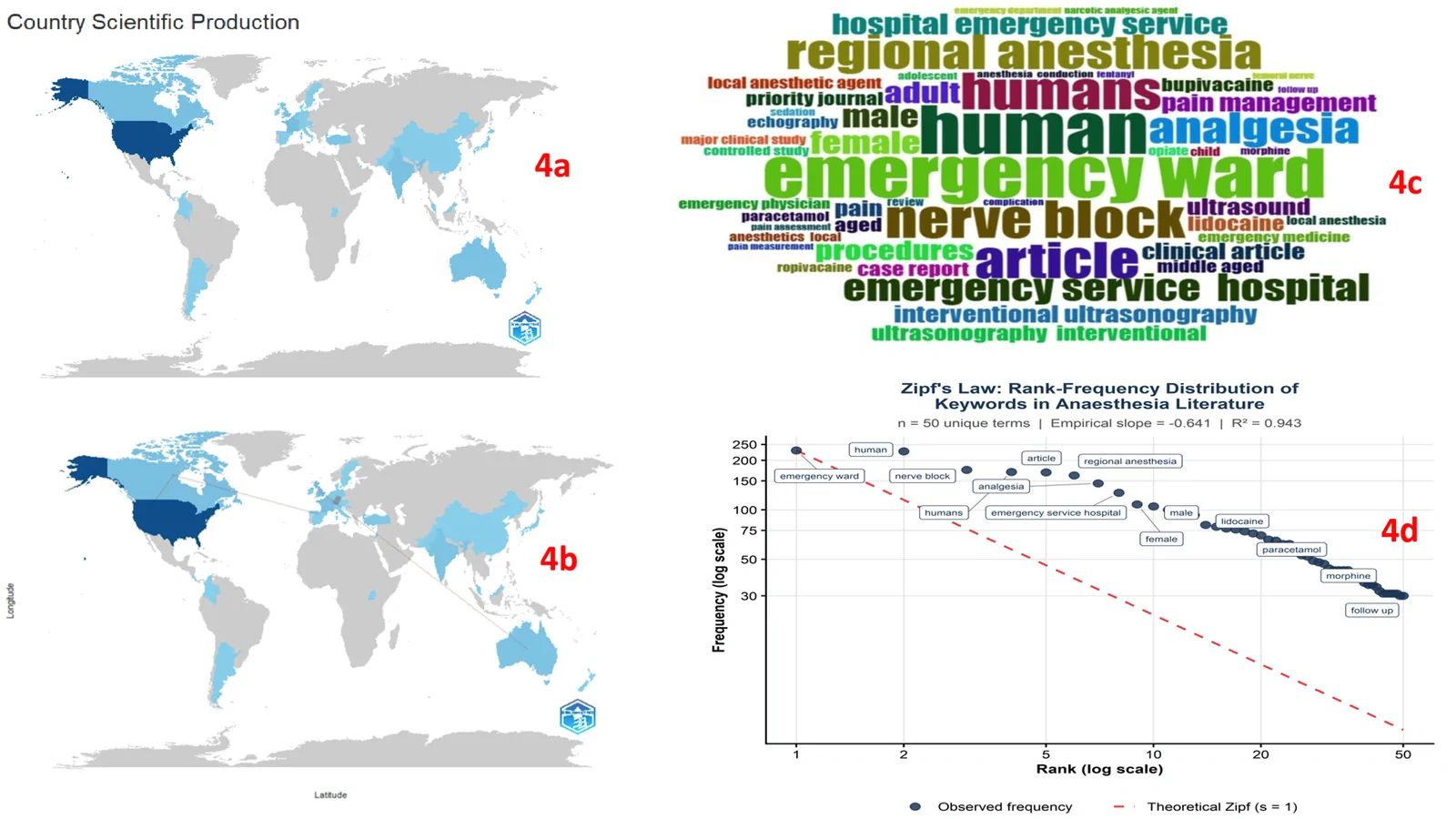
Geographical distribution, collaboration, co-citation, and keywords analysis a. Choropleth map visualizing volume of research output across the globe (Created using bibliometrix (R package), licensed under GNU GPL-3); b. Map visualizing international networks between contributing countries; c. Keyword WordCloud generated using Bibliometrix; d. Log-log rank-frequency plot of keywords used to assess conformity with Zipf's law. The x-axis represents keyword rank on a logarithmic scale, and the y-axis represents keyword frequency on a logarithmic scale. Red points indicate the ten most frequent keywords, and blue points indicate the remaining keywords.

The United States emerged as the global leader in both publication volume (n=94) and total citations (n=1297) (Figure 4). Other significant contributors included France, Australia, Canada, and Turkey (Figure 4a). The international collaboration map indicates active but largely regional clusters in North America, Europe, and Asia (Figure 4b, Table 5).

**Table 5:** Details of co-authorship and countries generated using the VOSviewer

Co-authorship and countries		
Country	Documents	Citations
Australia	11	147
Canada	15	206
France	16	59
Germany	5	4
India	11	71
Netherlands	6	57
Switzerland	7	17
Turkey	7	58
United Kingdom	17	198
United States	126	1840

Co-citation and Keyword Analysis

Co-citation analysis across 1,558 references identified Amini et al. [21] (2016) as the most frequently co-cited reference (n=12), with AJEM being the most co-cited source (Tables 6-8). Keyword visualization identified "regional anesthesia" and emergency-care terminology as prominent components of the literature (Figure 4c). After standardization, clinically interpretable themes centered on nerve blocks, analgesia, ultrasound guidance, and specific peripheral nerve or fascial plane techniques. The raw rank-frequency distribution used for Zipf analysis also contained generic indexing terms such as "human"; these terms were retained only for the mathematical rank-frequency assessment and were excluded from thematic interpretation (Figure 4d, Table 9).

**Table 6:** Co-citation and cited references generated using the VOSviewer

Co-citation and cited references		
Cited reference	Journal	Citations
Beaudoin FL et al., 2013 [12]	Academic Emergency Medicine	7
Amini R et al., 2016 [21]	Journal of Ultrasound in Medicine	12
Abou-Setta AM et al., 2011 [22]	Annals of Internal Medicine	6
Beaudoin FL et al., 2010 [23]	Journal of Emergency Medicine	8
Bhoi S et al., 2012 [24]	Journal of Emergencies, Trauma, and Shock	6
Morrison RS et al., 2016 [25]	Journal of the American Geriatrics Society	7
Ritcey B et al., 2016 [26]	Canadian Journal of Emergency Medicine	7
Stone MB et al., 2008 [27]	American Journal of Emergency Medicine	7
Wilson CL et al., 2017 [28]	AEM Education and Training	5
Wilson C et al., 2018 [29]	Annals of Emergency Medicine	5

**Table 7:** Co-citation and cited sources

Cited sources		
Source	Citations	Total link strength
American Journal of Emergency Medicine	51	36
Anesthesia and Analgesia	28	14
Annals of Emergency Medicine	49	34
Journal of Emergency Medicine	20	20
Regional Anesthesia and Pain Medicine	38	12

**Table 8:** Co-citation and cited authors

Cited authors		
Author	Citations	Total link strength
Andrew A.	14	18
Andrew J.	15	31
Benjamin W.	11	37
Friedman	11	37
Goldsmith A.	15	31
Herring AA.	13	18
Joseph M.	12	20
Knox H.	13	19
Michael A.	13	2
Neal JM.	12	20
Todd KH.	13	19

**Table 9:** Top ten keywords based on Bibliometrix

Rank	Terms	Frequency
1	emergency ward	230
2	human	227
3	nerve block	175
4	humans	170
5	article	169
6	regional anesthesia	162
7	analgesia	145
8	emergency service hospital	127
9	female	108
10	male	105

Discussion

This bibliometric analysis provides a comprehensive overview of the evolution and structural framework of research on RA in EM. The steady increase in scientific output over the last decade reflects growing academic attention to the use of RA techniques in the ED [30,31]. However, publication growth should not be equated with uniform clinical adoption or proven clinical benefit. Bibliometric output measures visibility and research activity, whereas implementation depends on training, supervision, equipment, governance, local protocols, and the capacity to manage complications. The collaborative authorship patterns suggest multidisciplinary involvement across EM, anesthesiology, and pain medicine. The concentration of publications in a small group of core EM journals highlights their role in disseminating procedural analgesia research, but it also suggests that the field has developed predominantly within EM rather than through balanced cross-specialty collaboration.

Citation analysis identified a small group of publications that have strongly influenced the intellectual structure of the field. The prominence of Beaudoin et al. [12] and Haines et al. [14] indicates sustained scholarly attention to ultrasound-guided femoral and fascia iliaca techniques in hip-fracture care. In bibliometric terms, their citation performance reflects their role as foundational references within this research network; it does not allow the present study to independently confirm their clinical conclusions. Similarly, the high citation count of Krauss et al. suggests that concerns about oligoanalgesia and multimodal pain management provided an important conceptual context for subsequent RA research. Citation counts must nevertheless be interpreted cautiously because they are affected by publication age, journal visibility, indexing, and the cumulative advantage of earlier papers, and they do not necessarily correspond to methodological quality or current clinical relevance.

The evolution of research themes, as indicated by the cleaned keyword analysis, suggests a shift towards ultrasound-guided peripheral nerve blocks and fascial plane blocks. The frequent appearance of erector spinae plane and fascia iliaca terminology identifies these techniques as areas of active investigation rather than demonstrating their comparative efficacy. The prominence of ultrasound-related terms is consistent with the wider integration of POCUS into emergency practice [32]. From an anesthesiology perspective, this thematic shift is important because it signals expanding use of RA techniques outside traditional operating-theatre and acute-pain-service pathways. It therefore raises questions regarding competency-based education, supervision, local anesthetic safety, rescue pathways, documentation, and shared governance between anesthesiology and emergency medicine.

Despite the global relevance of effective pain management, research remained geographically concentrated, with the United States leading publication volume and citations. This dominance may reflect established academic infrastructure, access to ultrasound technology, research funding, mature EM training systems, and the representation of North American journals within Scopus. International collaboration was low (9.9%), and most publications arose from single-country efforts. This concentration limits the external applicability of the research landscape because feasibility, training requirements, block selection, and safety systems may differ across resource settings. Multicenter and multinational collaboration is therefore needed not merely to increase publication counts, but to test whether implementation models and outcome measures are transferable across healthcare systems.

Implications for Clinical Practice and Future Research

The findings of this study underscore the increasing integration of RA into multimodal analgesia strategies within emergency care. Systematic reviews have demonstrated that techniques such as fascia iliaca and femoral nerve blocks provide highly effective analgesia while reducing opioid requirements [3,33,34]. The clinical value of this bibliometric analysis lies in identifying where evidence generation is concentrated, which techniques receive sustained attention, and where collaboration and outcome reporting remain limited. The analysis does not establish dose, indication, efficacy, or complication rates and should not be interpreted as a clinical practice guideline. For anesthesiologists, however, the observed growth of emergency physician-performed RA has practical implications for education, credentialing, multidisciplinary protocols, local anesthetic systemic toxicity preparedness, quality assurance, and referral pathways. The prominence of hip-fracture blocks, brachial plexus blocks, and newer fascial plane techniques may help prioritize areas for joint training and prospective evaluation, while the limited international network highlights the need for harmonized definitions and core outcomes.

A recent clinical review has further validated the feasibility and effectiveness of ultrasound-guided RA performed directly by emergency physicians [35]. However, despite this accumulating evidence, implementation in routine clinical practice remains inconsistent globally, revealing a significant ‘research-to-practice’ gap. Future research should move beyond descriptive reports and small single-centre studies towards prospective multicenter designs that evaluate clinically relevant endpoints. Priorities include block success, opioid exposure, procedural delay, patient satisfaction, functional recovery, complications, rescue analgesia, length of stay, training competency, workflow integration, and cost-effectiveness. Collaboration between anesthesiologists and emergency physicians will be particularly important for developing competency-based curricula, safety standards, and reproducible implementation pathways. These recommendations arise from gaps revealed by the bibliometric structure of the field; they should be tested through clinical research rather than assumed from publication trends alone.

Study Limitations

This study has several limitations. First, the analysis was restricted to Scopus. Although Scopus provides the structured citation and affiliation metadata needed for bibliometric mapping, its journal coverage and indexing policies may favor languages, regions, and publication types. Studies indexed only in PubMed/MEDLINE, Web of Science, or regional databases may therefore have been omitted, and country, institutional, or citation rankings may differ if another database is used. Second, citation metrics favor older publications and may also be influenced by journal visibility, self-citation, review-article citation patterns, and database indexing rather than clinical importance. Third, bibliometric analysis evaluates research quantity, connectivity, and visibility but does not assess the methodological quality of individual studies or establish clinical efficacy. Fourth, variation in terminology may affect keyword mapping. Although spelling variants and synonymous terms were standardized, residual inconsistencies in author keywords and indexing terms may remain. Finally, bibliometric relationships are sensitive to data-cleaning choices and threshold settings. The findings should therefore be regarded as a reproducible map of the Scopus-indexed literature rather than a comprehensive assessment of all evidence on RA in emergency care.

## Conclusions

Research on RA in EM has expanded markedly over the past decade, with scientific activity concentrated in a limited number of countries, institutions, authors, and journals. Ultrasound-guided peripheral nerve and fascial plane blocks have become prominent research themes, but bibliometric prominence does not establish clinical superiority or routine implementation. For anesthesiology, the findings define opportunities for multidisciplinary leadership in training, safety governance, outcome standardisation, and multicentre evaluation of RA beyond the operating theatre. Broader international collaboration and clinically focused research are required to determine how these evolving research priorities can translate into safe, effective, and context-appropriate emergency pain care.
